# Localised Electrochemical Impedance Spectroscopy of Gold Nanoparticles Labelled Antibodies Probed by Platinum Microstructured Ultramicroelectrode

**DOI:** 10.3390/ma17061339

**Published:** 2024-03-14

**Authors:** Antanas Zinovicius, Inga Morkvenaite-Vilkonciene, Arunas Ramanavicius

**Affiliations:** 1 Department of Mechatronics, Robotics and Digital Manufacturing, Vilnius Gediminas Technical University, 10105 Vilnius, Lithuania; antanas.zinovicius@vilniustech.lt; 2 Department of Nanotechnology, State Research Institute Centre for Physical Sciences and Technology, 02300 Vilnius, Lithuania; 3 Department of Electrical Engineering, Vilnius Gediminas Technical University, 10105 Vilnius, Lithuania; 4 Department of Physical Chemistry, Faculty of Chemistry and Geosciences, Vilnius University, 03225 Vilnius, Lithuania

**Keywords:** scanning electrochemical impedance microscopy, SEIM, ultramicroelectrode, electrode modification, electrochemical immunosensor, transducer, reusable transducer

## Abstract

This research is focused on enhancing the capabilities of scanning electrochemical impedance microscopy (SEIM) for detecting gold nanoparticle-labelled antibodies using electrochemically modified platinum ultramicroelectrode. The primary objective was to address the high resistance issue encountered in previous measurements with SEIM via the utilization of SEIM probes based on micro-electrodes modified by platinum microstructures, which improved the sensitivity and precision of the detection of targeted biomolecules. The modified probe resulted in a lowered charge transfer resistance by over ten times and a decrease in detection to around 100 fg/mL. We suggest potential applications in various biotechnological and biomedical fields, with future research expected to further refine this technique.

## 1. Introduction

Early and effective disease prevention depends on the availability of sensitive and simple-to-use detection tools for critical disease markers. Immunosensors are an emerging tool in the fields of medical diagnostics and biological research for several compelling reasons [1]. These specialised devices exhibit a remarkable combination of specificity and sensitivity, thus enabling the precise detection and quantification of specific molecules, such as proteins, viruses, and pathogens [2,3,4,5]. This level of accuracy is crucial for identifying disease markers, tracking disease progression, and monitoring various biological processes. Moreover, immunosensors are renowned for their ability to deliver rapid results, making them invaluable in emergencies and point-of-care testing, where swift decisions regarding patient care are vital [6,7]. This not only enables timely intervention and care for the patient, but also the ability to monitor important biological processes.

The integration of advanced nanotechnologies into the field of biosensors has significantly broadened the scope of disease detection and monitoring, offering a sophisticated approach to enhance the sensitivity and performance of such devices. Notably, innovative nanomaterials and biocomposites have become a cornerstone in the development of highly efficient biosensors. Nanomaterials, including nanoparticles, nanowires/nanorods, nanocrystals, nanotubes, dendrimers, and quantum dots, can be tailored to suit specific applications via the manipulation of the shape, form, and size of the structure [8,9]. Their unique catalytic, magnetic, optical, mechanical, and electric properties are attributed to their surface characteristics, small size, or macroscopic tunnelling effects, which play a crucial role in their functionality [10].

The exceptional high surface-to-volume ratio of these nanomaterials facilitates efficient conjugation with biomolecules or polymers, while retaining their catalytic activity. This property is pivotal for the promotion of electron transfer between the redox centre and the electrode surface, thus enhancing the charge transfer in electrochemical biosensors [11]. Given these advancements, nanomaterials have become indispensable in constructing electrochemical biosensors, which are integral to the early and effective prevention of disease.

Immunosensors in particular have gained prominence in medical diagnostics and biological research due to their unparalleled specificity and sensitivity. These specialised devices are capable of detecting and quantifying specific molecules, such as proteins, viruses, and pathogens, with remarkable precision. Due to these reasons, the enzyme-linked immunosorbent assay method is considered the gold standard. However, the processes of preparing, purifying, conjugating to another biomolecule, and storing enzymes are often costly and require significant amounts of time. Moreover, enzyme functionality is prone to being affected by external factors such as temperature, pH levels, and mechanical stress. To address these challenges, researchers are searching for an alternative catalytic label. It was found that nanoparticles, especially those made from noble metals like gold, platinum, and palladium, demonstrate catalytic activity similar to enzymes when performing oxidation, reduction, hydrogenation, or dehydrogenation processes [12]. These nanoparticles can be used as an excellent label for creating innovative non-enzymatic immunosensors because they are more stable, can function across a wider range of temperatures and pH levels, and have similar preparation processes to traditional biomolecules.

Since the introduction of immunosensors, a variety of different sensing techniques have been reported, and, recently, electrochemical immunosensing has gained considerable interest [13]. This has relatively simple instrumentation, which can be scaled down at the circuit board level [14,15]. This simplifies the creation of disposable devices for point-of-care diagnostics, offering high sensitivity with small sample consumption [16,17]. Electrochemical immunosensors that use electrochemical impedance spectroscopy (EIS) have gained prominence due to their exceptional sensitivity and specificity in biomolecule detection, ensuring precise and reliable results [15,18,19]. A key advantage is their ability to achieve label-free detection, thus simplifying the assay process and reducing costs [20]. Furthermore, these sensors enable the real-time monitoring of biomolecular interactions, providing valuable insights into binding kinetics and affinity [21,22]. Typical immunosensors based on EIS rely on the physical blocking of the electrode surface. As antigens bind to antibodies, impedance is introduced to the circuit, resulting in a decrease in signal strength [18,19]. However, EIS in bulk solutions may not provide detailed information regarding the targeted area of interest within the sample.

Scanning electrochemical microscopy (SECM) is a versatile surface characterisation technique that merges microscopy and electrochemistry, thus enabling the study of materials and interfaces at high spatial resolutions. SECM is one of the scanning probe microscopy techniques using ultramicroelectrodes (UMEs) as a probe [23]. This allows the probing of electrochemical processes at the micro- or nano-scale, providing spatially resolved information regarding surface electrochemical reactivity and topography [24,25]. SECM offers the unique ability to locally analyse corrosion, material interfaces, and biological systems.

To probe the highly localised regions of the sample with a high level of sensitivity, a combination of scanning electrochemical microscopy and EIS (SEIM) can be used. SEIM offers the ability to measure the entire electrochemical impedance spectra across all points of interest of in 3D spaces. Thus, SEIM has a heightened sensitivity to small changes in impedance as measurements can be taken near the surface of interest. Additionally, using SEIM provides high spatial resolution, thus making it a superior choice when examining microelectrodes [26,27]. Although SEIM is a powerful method for localised surface studies due to the use of ultramicroelectrodes and the small probe-to-sample distance, high resistance can be registered [28], which results in several technical problems. One significant drawback is the low signal-to-noise ratio, meaning the measured signal then becomes weak compared to background noise or interfering signal, making it rather challenging to obtain accurate and reliable data. Additionally, extremely high resistance can influence the accuracy of these measurements by becoming comparable to or even exceeding the absolute high-impedance of the potentiostat and cables [29]. This situation can introduce significant distortions to the measurements. To solve this high resistance problem, the conductive surface of the probe could be modified by forming microstructures on the surface of electrodes. This solution could lower the resistance without compromising the advantages of localised measurements.

The aim of our research was to apply the localised EIS for the detection of gold nanoparticle (AuNPs)-labelled antibodies by increasing system resistance using platinum microstructured probes. For this, different concentrations of goat anti-human immune globulin G (IgG) labelled by 6 nm gold nanoparticles, which were immobilised on non-conductive surface, were analysed using SEIM with non-modified and platinum-modified microstructure probes.

## 2. Materials and Methods

### 2.1. Materials

The phosphate buffer solution (PBS) was prepared using tablets (Sigma-Aldrich, St. Louis, MI, USA). A mediator solution of 0.4 M K_3_Fe(CN)_6_/K_4_Fe(CN)_6_ (Merck, Darmstadt, Germany) and 1 M glucose (Carl Roth GmbH & Co., Karlsruhe, Germany) were prepared in the PBS to avoid any pH change when the mediator and the substrate were added to the main solution. The glucose solution was left to mutarotate for 24 h before use. Furthermore, a solution of 2 mM K_2_PtCl_6_ (Sigma-Aldrich, St. Louis, MI, USA) was prepared in 0.5 H_2_SO_4_ (Merck, Darmstadt, Germany), where all the dissolved oxygen was removed using bubbling nitrogen for 5 h.

### 2.2. Antibody Immobilization

Goat anti-human IgG labelled with 6 nm gold nanoparticles (IgG-AuNP) were obtained from Abcam (Cambridge, UK). Different concentrations ranging from 10 µg/mL to 100 fg/mL were prepared in the PBS. The glass slide was washed with 98.5% ethanol solution (Vilniaus Degtinė, Vilnius, Lithuania), rinsed with deionised water, and left to dry under laminar flow. The clean glass slide was then kept in a closed vessel containing a 25% glutaraldehyde solution for 15 min to form the first layer of glutaraldehyde. Afterwards, a 0.5 µL drop of the IgG-AuNP solution was deposited onto the prepared glutaraldehyde layer and left to dry under laminar flow. Then, the sample was exposed to the 25% glutaraldehyde solution for an additional 15 min to crosslink the deposited antibodies. 

### 2.3. Measurements

Prior to the experiments, the ultramicroelectrode (UME) underwent a cleaning electrochemical process according to the manufacturer’s guidelines. This involved cycling in 0.5 M H_2_SO_4_ within a potential range of 0–1.2 V vs. Ag/AgCl_(3M/L KCl)_, followed by rinsing with deionised water, ethanol, and deionised water once more. For the scanning electrochemical microscope (Sensolytics, Bochum, Germany), a 10 µm diameter platinum UME with an R_g_ value of 10 was used as a probe. An Ag/AgCl in 3 M KCl electrode (MetrohmAG, Herisau, Switzerland) was used as the reference electrode, alongside a platinum wire with a substantial surface area (which is at least 100 times larger than that of the UME). The distance between every electrode was set at 10 mm.

At the beginning of each experiment, cyclic voltammetry measurements were performed within the range of −600 mV to 600 mV with a scan rate of 10 mV/s and a step of 10 mV. This was to evaluate the UME conditions, seeking the precise peak current potential for further measurements. 

All electrochemical measurements were carried out in a solution containing 1 mM of K_3_[Fe(CN)_6_]/K_4_[Fe(CN)_6_] and 50 mM of glucose. The sample surface was probed by approaching it with a 1 µm step and a speed of 1 µm/s, with a 10 ms wait time. After the probe was positioned at the desired location, EIS measurements were performed.

**Figure 1:**
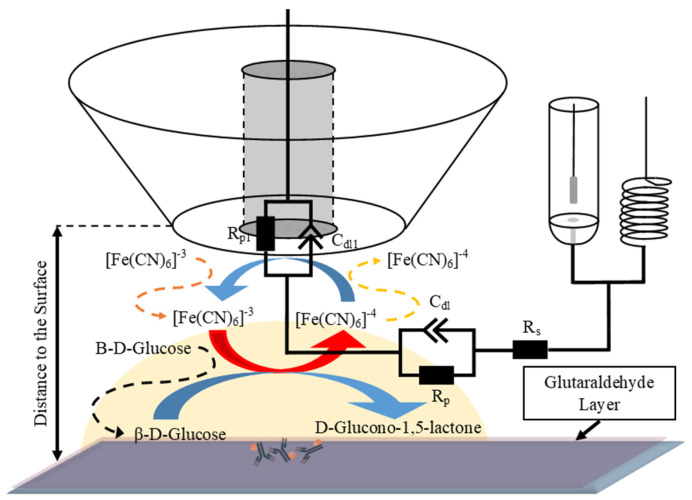
A schematic representation of the feedback mode for the electrochemical impedance microscopy measurements of the IgG-AuNP-modified surface in the presence of glucose and potassium ferrocyanide (K_4_[Fe(CN)_6_]), which serves as a redox mediator.

Impedance measurements were performed within the frequency range of 100 mHz to 50 kHz (logarithmical change) with a root mean square amplitude of 10 mV, a potential bias of +400 mV, and the distance between the sample surface and the tip of the probe being 2 µm. The obtained data from experimental measurements were then fitted to an equivalent circuit, as depicted in Figure 1, which depicts the system of interest. 

The mathematical impedance dependence is expressed as

(1) $$Z=\frac{Z_{Cdl}\cdot \left(R_{p}\right)}{Z_{Cdl}+\left(R_{p}\right)}+R_{s}$$

where $Z_{Cdl}$ is the double layer impedance (Equation (2)), $R_{p}$—the charge-transfer resistance, and $R_{s}$—the ohmic resistance of the solution.

Double layer impedance is expressed as a constant phase element as follows:

(2) $$Z_{Cdl}=\frac{1}{Q\;{\left(j\omega \right)}^{\alpha }}$$

where *Q* represents the capacitance of a CPE with an *α* of 1, *j*—the imaginary unit, *ω*—the angular frequency, and *α* represents the angle by which the CPE impedance is rotated.

### 2.4. Ultramicroelectrode Modification

To reduce electrochemical impedance during the measuring process, the UME was modified using electrochemically forming platinum structures on the conductive part of the electrode. The UME was immersed in a 2 mM K_2_PtCl_6_ solution, and cyclic voltammetry was applied within a range of −200 mV to 1.3 V, with a step size of 50 mV for 10 cycles. Nucleation was induced at 1 V. To ensure stable conditions, a nitrogen atmosphere was used while performing modification. 

The same procedure was used to form platinum structures on a 3 mm diameter graphite electrode (Sigma-Aldrich, Steinheim, Germany) for scanning electron microscopy analysis.

### 2.5. Scanning Electron Microscopy Analysis

The investigation of the electrochemically formed platinum structure surface was conducted using a Helios Nanolab 650 scanning electron microscope (FEI, Eindhoven, The Netherlands) with a voltage of 2 kV and a current of 0.4 nA at a magnification of 10,000 times.

## 3. Results

This assessment of catalytic activity offers insights into the intricate biological mechanisms of SEIMs. To study these processes at a localised level, researchers have employed SEIM using ultramicroelectrodes. In previous studies conducted by our research group, a promising method utilizing the redox-competition mode for scanning electrochemical impedance microscopy was introduced [28]. However, the implementation of this method is not without its challenges. One of the most significant obstacles arises from the inherent characteristics of UMEs used in SEIM. Due to the small size of the probe and the necessity of being in close proximity to the sample surface, impedance and charge transfer resistance often soared to values exceeding a giga-ohm. This phenomenon is attributed to the limited area of the electrode, which, while beneficial for achieving high spatial resolution, also restricts the flow of the current, exacerbating impedance levels. 

### 3.1. Ultramicroelectrode Comparison

**Figure 2:**
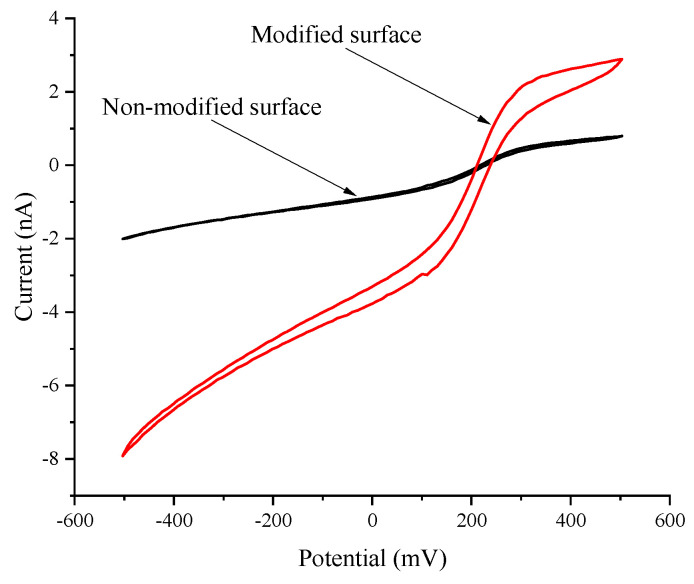
Cyclic voltammograms of modified and non-modified platinum ultramicroelectrodes (diameter—10 µm). Measurements were performed in a phosphate-buffered solution with the 1 mM K_3_[Fe(CN)_6_]/K_4_[Fe(CN)_6_] and 50 mM of glucose.

In attempts to mitigate this issue without compromising localisation, ultramicroelectrodes were electrochemically modified via forming platinum microstructures on the conductive part of the electrode. Platinum was selected to extend UMEs due to its exceptional electrical conductivity, its stability under various chemical conditions, and its biocompatibility. The modification process aimed to increase the electrode’s surface area by forming uniform electrodes on the surface. This is crucial for obtaining accurate and reproducible measurements in electrochemical sensing applications. To assess the modification, cyclic voltammograms were recorded (Figure 2). 

The results demonstrated a 4.3-fold increase in the current generated near the modified surface, which is significant when compared to the non-modified ultramicroelectrode at the anodic peak potential. Formed structures facilitate higher charge transfer rates, leading to an increased current output. Additionally, the observed increase in current at the anodic peak potential serves as a clear indicator of the successful integration of platinum microstructures with the electrode’s pre-existing framework.

Furthermore, we employed scanning electron microscopy to morphologically evaluate the newly formed platinum microstructures. This analytical technique allowed for the high-resolution visualization of the microstructures’ shapes, sizes, and distribution patterns across the electrode surface, thus offering invaluable insights into the physical characteristics of the modifications. To accommodate the physical constraints imposed by SEM, platinum microstructures were formed on the surface of the graphite electrode, providing not only a suitable platform but also a higher surface area to observe.

**Figure 3:**
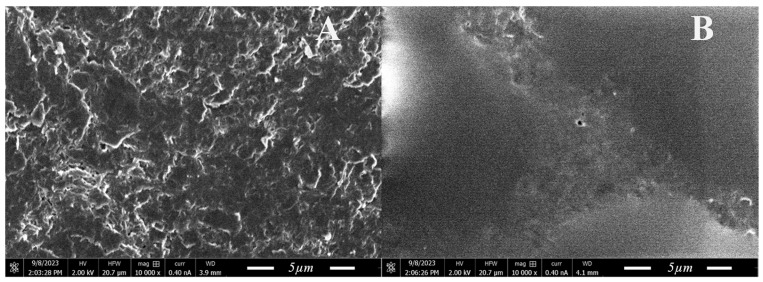
Scanning electron microscopy images under 10,000 times magnification of (**A**)—polished graphite electrode; (**B**)—Graphite electrode modified by platinum microstructures.

Figure 3 depicts SEM images of non-modified and modified surfaces at a magnification of 10,000 times. The examination of surface morphology unveiled the presence of platinum structures atop the graphite electrode. These microstructures exhibited a non-uniform convex shape form with diameters varying significantly across a range from 2.5 to 35 µm. The variation in size and the convex shape of the platinum structures are critical observations, as they indicate the non-homogeneous nucleation and growth of platinum during the modification process. 

This observation suggested that similar morphological changes would have occurred on the conductive surface of the ultramicroelectrode. The introduction of such platinum microstructures on the electrode’s surface could potentially have a significant impact on its electrochemical behaviour, particularly in terms of reducing impedance resistance during any subsequent measurements.

### 3.2. Local Electrochemical Impedance Measurement

**Figure 4:**
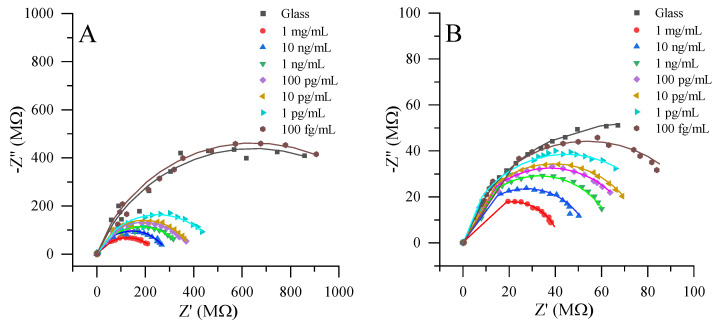
Nyquist plots observed at a 2 µm distance from the sample. (**A**). Non-modified platinum ultramicroelectrode; (**B**). Modified platinum ultramicroelectrode, near different concentrations of immobilised Ab-AuNP, starting from 1 mg/mL to 0 mg/mL. Measurements were performed in the phosphate-buffered solution with the 1 mM K_3_[Fe(CN)_6_]/K_4_[Fe(CN)_6_] and 50 mM glucose.

In the following sections, we delve into the local electrochemical impedance measurements, comparing the performances of both the modified and non-modified ultramicroelectrodes. For both the modified and non-modified UMEs, a common experimental setup was employed, as depicted in Figure 1. The inclusion of antibodies conjugated with gold nanoparticles facilitated enzyme-mimicking reactions. This approach enables the reduction of [Fe(CN)_6_]^3−^ to [Fe(CN)_6_]^4−^, which could subsequently be reversed at the ultramicroelectrode surface via oxidation. This method allowed the ultramicroelectrode to record the formation of [Fe(CN)_6_]^4−^, directly correlating it with the exhibited catalytic activity of gold nanoparticles within the IgG-AuNP conjugate. Electrochemical impedance spectroscopy spectra were collected at various concentrations of the IgG-AuNP conjugate, ranging from 1 mg/mL to 100 fg/mL (Figure 4). This extensive range allowed for a detailed analysis of the electrode’s sensitivity and its detection limit for catalytic activity. A glass substrate served as a control in these experiments, providing a baseline for comparisons of the impedance characteristics of the sample.

The inclusion of EIS in this study offers a comprehensive understanding of the dynamic electrochemical processes taking place at the electrode surface, particularly the kinetics of the redox reactions facilitated by the IgG-AuNP conjugate. By examining the impedance changes across various concentrations of the conjugate, the research sheds light on the efficiency of the modified and non-modified electrodes in facilitating and detecting these biochemical reactions.

As anticipated, the impedance measurements using the non-modified ultramicroelectrode revealed significantly higher resistance levels when compared to the modified counterpart. This distinction in resistance highlights the effectiveness of the surface modifications applied to the UME in enhancing its electrochemical performance. Both systems exhibited changes in impedance across the entire concentration range, thus demonstrating the sensitivity of this electrochemical method in monitoring the catalytic activity facilitated by IgG-AuNP. 

**Figure 5:**
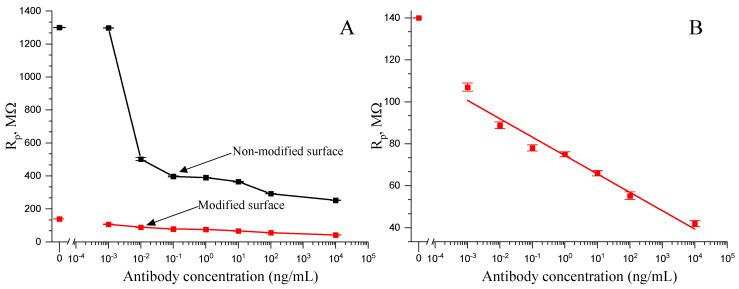
Charge transfer resistance logarithmic dependency on immobilised Ab-AuNP concentrations. (**A**). Comparison between modified and non-modified electrodes; (**B**). Results from the modified electrode fitted to the linear model. Measurements were performed in a phosphate-buffered solution with the 1 mM K_3_[Fe(CN)_6_]/K_4_[Fe(CN)_6_] and 50 mM glucose.

To quantitatively assess the charge transfer resistance, which is a key parameter in evaluating the efficiency of electrochemical processes at the electrode surface, the experimental data were analysed using the Randles circuit model (Equations (1) and (2)). This approach facilitated the extraction of charge transfer resistance parameter, which directly correlates with the ease of electron transfer across the electrode interface. The comparison of the charge transfer resistance between non-modified and modified UMEs highlighted a significant difference in the electrochemical performance of the system (Figure 5A). The charge transfer resistance observed for the non-modified electrode, which exceeded 1 GΩ, was indicative of a substantial barrier to the electron transfer across the electrode–analyte interface. Such high resistance levels can detrimentally affect the kinetics of electrochemical reactions, thereby limiting the sensitivity and efficiency of the electrode in analytical applications. In contrast, the use of the modified electrode resulted in a charge transfer resistance of just over 100 MΩ, representing a more than ten-fold reduction in resistance. This decrease in charge transfer resistance using modified electrode signifies an improvement in the electrochemical reaction kinetics, thus enabling higher reliability and sensitivity levels. 

Furthermore, measurements further revealed that the utilization of the modified electrode not only significantly reduced the charge transfer resistance, but also improved the consistency and precision of measurements. Specifically, the modified electrode demonstrated lower deviation levels when compared to the non-modified electrode, indicating enhanced stability and reliability in electrochemical measurements. This improvement is particularly important in applications requiring high precision and accuracy, such as in the quantification of catalytic activities in complex biological samples. 

Moreover, the modifications applied to the electrode substantially extended its linear response range. While the non-modified electrode exhibited a linear detection range up to 1 pg/mL, the modified electrode showed a remarkable extension of this range down to 100 fg/mL. This extension of the linear range is critical for the detection of extremely low analyte concentrations, enhancing the electrode’s applicability in sensitive analytical assays, where the precise quantification of trace levels of substances is required. 

In addition to extending the linear range, the modified system exhibits a lower limit of detection (LOD), reaching 100 fg/mL. This represents a significant improvement over the non-modified electrode, which had an LOD of 1 pg/mL. The reduced LOD in the modified electrode system underscores the enhanced sensitivity afforded by the electrode modifications, making it a valuable tool for catalytic activity assessments in biological samples.

Table 1 represents a comparison of a typical impedimetric immunosensor to the SEIM immunosensor. The constructed immunosensor demonstrates simple electrode modification without antibodies obstructing the electrode itself, thus retaining its ability to be reused after washing. Additionally, showing one of the longest linear ranges of 0.001–10^3^ ng/mL when compared to other immunosensors, demonstrates the broad scope of potential methods and applications. Moreover, the most efficient analysis time of just 20 min was demonstrated. 

## 4. Conclusions

These findings represent a significant advancement in the field of SEIM, offering a promising method for investigating biological samples with higher levels of precision and accuracy. The successful modification of ultramicroelectrodes demonstrates the potential for enhancing the capabilities of this technique, which can have far-reaching implications in various scientific and biomedical applications. The initial high resistance (1.29 GΩ) encountered with non-modified ultramicroelectrodes was significantly reduced following the successful modification, which led to a more than ten-fold decrease in the charge transfer resistance, reaching up to 120 MΩ. The developed system is more competitive when compared to typical impedimetric immunosensors. It requires simpler preparation procedures, utilising immobilisation on inexpensive substrate like glass, all while maintain an ample linear range (0.001–10^3^ ng/mL), thus achieving a low limit of detection (0.001 ng/mL), and minimising the time needed for analysis to just 20 min. Further research focusing on this approach holds the potential to unlock new insights into the catalytic behaviour of biological samples, ultimately advancing our understanding of complex biological processes.

## Figures and Tables

**Table 1:** Electrochemical impedimetric immunosensors.

Analyte	Used Electrode	Linear Range	LOD	Analysis Time, min	Ref.
Human IgG	platinum-based UME modified with platinum microstructures	0.001–10^3^ ng/mL	0.001 ng/mL	20	This research
Tetracycline	eight gold microelectrodes functionalised with 4-aminophenylacetic acid, the structure of magnetic nanoparticles coated with poly (pyrrole-co-pyrrole-2-carboxylic acid and crosslinked with specific polyclonal antibody for tetracycline	0.0001–1 ng/mL	0.0012 ng/mL	60	[30]
Microalbuminuria	carbon interdigitated microelectrode modified with gold nanocrystals with trapped nanoprobes (polystyrene nanoparticle core with silver nano-shells covalently conjugated to HSA antibodies)	30–300 μg/mL	30 μg/mL	-	[31]
Dengue virus	screen-printed carbon electrodes coated with oxidised bovine serum albumen and functionalised by anti-non-structural monoclonal antibodies	1–200 ng/mL	0.3 ng/mL	30	[32]
Mucin 4	carbon-based screen-printed electrode functionalised with phenylacetic acid with immobilised human partial recombinant MUC4 protein and mouse monoclonal antibody	1–15 μg/mL	0.33 μg/mL	30	[33]
Cry1Ab	indium tin oxide electrodes modified by 3-aminopropyltrimethoxysilane monolayer with covalently immobilised Anti-Cry1Ab polyclonal antibodies	1–10 ng/mL	0.37 ng/mL	60	[34]
Aflatoxin B1	gold electrode modified by 1,6-hexanedithiol and coated with gold nanoparticles and functionalised with Aflatoxin B1-bovine serum albumin (BSA)	0.08–100 ng/mL	0.05 ng/mL	50	[35]
1-Aminohydantoin	glassy carbon electrode modified with sol-gel-monoclonal anti-body against 1-Aminohydantoin	2–10^3^ ng/mL	2 ng/mL	160	[36]

## Data Availability

Data are contained within the article.
